# ExacTrac Dynamic Surface-Guided Setup Versus Tattoo-Based Positioning in Breast Cancer Radiotherapy: A Retrospective Comparison of Setup Accuracy and Preparation Time

**DOI:** 10.3390/cancers18203375

**Published:** 2026-10-19

**Authors:** Bilal Krayim, Adar Yaacov, Mohammad Abu Myala, Muhammad Awad, Dolev Kahla, Basel Afifi, Basil Dabbah, Roni Gillis, Albert Grinshpun, Areen Abu Ramila, Amir Abramovich, Hila Rosenfield, Nir Honig, Sion Koren, Sharon Rigler, Raphael Pfeffer, Aaron M Allen

**Affiliations:** Helmsley Cancer Center-Shaare Zedek Medical Center, The Hebrew University of Jerusalem, Shmuel Bait 12, Jerusalem 9103102, Israelmuhamadabu@szmc.org.il (M.A.M.);

**Keywords:** surface-guided radiation therapy (SGRT), ExacTrac Dynamic, breast cancer, breast radiotherapy, patient setup accuracy, image-guided radiotherapy (IGRT), tattoo-free positioning, setup reproducibility, treatment workflow efficiency

## Abstract

**Background/Objectives:** Accurate and reproducible patient positioning is essential in breast cancer radiotherapy to ensure target coverage while minimizing exposure to surrounding healthy tissues. Conventional tattoo-based alignment remains widely used but has inherent limitations, including variability, potential setup errors, and negative psychological impact from permanent markings. Surface Guided Radiation Therapy (SGRT) using ExacTrac Dynamic (ETD) offers a non-invasive, real-time alternative that may improve workflow efficiency and patient experience. This study evaluated the accuracy and efficiency of ETD compared with conventional tattoo-based setup in breast-only radiotherapy. **Methods:** We conducted a retrospective comparative cohort analysis of patients treated for breast cancer at Shaare Zedek Medical Center between December 2022 and December 2024. Forty-nine patients (245 fractions) were positioned using SGRT with ETD, while a control cohort of 20 patients (100 fractions) underwent conventional tattoo-based alignment; all patients received breast-only irradiation (26 Gy in 5 fractions) in deep inspiration breath-hold (DIBH), and all received daily kilovoltage (kV) imaging, which served as the reference. Six-dimensional pre-correction setup deviations and treatment preparation times were recorded. Because fractions from the same patient are correlated, the primary analysis was performed at the patient level (random-intercept model, equivalent to comparison of patient means in this balanced design), with patient-level (cluster) bootstrap confidence intervals, Bonferroni correction across the six axes, and rank-biserial effect sizes. **Results:** Setup errors were strongly clustered within patients (intraclass correlation up to 0.75). At the patient level, absolute setup errors did not differ significantly between cohorts on any axis (all *p* ≥ 0.11), nor did composite translational (*p* = 0.27) or rotational (*p* = 0.70) errors. SGRT showed a mean vertical offset of −5.1 mm relative to control (95% CI −8.9 to −1.3 mm; *p* = 0.013, Bonferroni-adjusted *p* = 0.078). Clinically significant pre-correction deviations (≥5 mm) occurred in 52–65% of fractions in the vertical and longitudinal directions in both cohorts. Mean preparation-for-treatment time was shorter with SGRT (11.0 vs. 13.9 min; median 10.0 vs. 12.5 min; *p* = 0.036). **Conclusions:** Initial setup with ExacTrac Dynamic showed no statistically significant difference in setup accuracy compared with tattoo-based setup and shortened preparation time but was associated with a vertical offset. Pre-correction errors were large with both methods, so daily image guidance remains necessary. Because backup tattoos were placed in all patients and equivalence was not formally tested, these results support—but do not establish—tattoo-free daily setup. Prospective, multicenter studies are warranted to validate these findings and assess long-term clinical outcomes.

## Simple Summary

Radiotherapy for breast cancer usually requires permanent skin tattoos so patients can be lined up precisely at every treatment. These tattoos can cause lasting distress and body-image concerns. Surface Guided Radiation Therapy (SGRT) is a camera-based technology that can position patients without relying on tattoos. We compared a newer SGRT system, ExacTrac Dynamic, with standard tattoo-based setup in 69 breast cancer patients treated at our center. We measured how accurately each method positioned patients and how long setup took. When each patient was analyzed as a unit, setup accuracy did not differ significantly between the two methods in any direction, although the camera-based setup tended to be about 5 mm off in the up–down direction compared with the X-ray reference. Setup errors before X-ray correction were large with both methods, confirming that daily X-ray checks remain necessary. The camera-based setup shortened preparation time by about three minutes per treatment. Because patients in this study still received backup tattoos, these findings support, but do not yet prove, that daily setup for breast radiotherapy can be performed without permanent tattoos.

## 1. Introduction

Accurate and reproducible patient positioning is a cornerstone of modern radiation therapy, ensuring that the prescribed dose is delivered precisely to the target while minimizing exposure to adjacent organs at risk [1]. In breast cancer radiotherapy, conventional setup methods typically rely on permanent skin tattoos aligned with laser systems. While effective, these methods are not without limitations: they are prone to variability in reproducibility, introduce the potential for setup errors, and can have a lasting negative psychological impact on patients because of permanent skin markings [2,3].

Surface Guided Radiation Therapy (SGRT) has emerged as a promising non-invasive alternative to skin tattoos [4]. By using three-dimensional optical surface imaging, SGRT continuously compares the patient’s external anatomy with the reference model from the treatment plan. In phantom studies, surface imaging has shown sub-millimeter registration accuracy, and it allows real-time monitoring of patient motion throughout treatment delivery [5]. Such capabilities are particularly advantageous for advanced techniques such as Deep Inspiration Breath Hold (DIBH), where accurate timing and reproducibility are critical to reducing cardiac and pulmonary irradiation in left-sided breast cancer [6,7].

Beyond technical performance, SGRT offers potential advantages in patient experience. Avoiding permanent tattoos may spare patients psychological distress and body image concerns associated with lifelong skin markings [3,8]. Furthermore, SGRT enables monitoring of intra-fraction motion and has been reported to reduce the need for repeated imaging and to streamline treatment workflows [9,10].

Although prior studies have demonstrated the feasibility and accuracy of SGRT in breast cancer radiotherapy [9], there is still limited evidence regarding the clinical performance of newer integrated platforms such as ExacTrac Dynamic (ETD), especially in the context of routine breast-only treatments [11]. These systems combine surface imaging with real-time monitoring, offering the potential for improved reproducibility, workflow efficiency, and treatment safety.

Emerging evidence has begun to address the performance of ETD specifically in breast radiotherapy. Da Silva Mendes et al. demonstrated submillimeter geometric agreement between the surface-imaging and stereoscopic X-ray components of ETD and cone-beam computed tomography (CBCT) in a clinical workflow evaluation [12]. Katayama et al. reported a high correlation between ETD-derived surface tracking and portal-imaging motion waveforms in 16 breast cancer patients treated under free-breathing conditions, although longitudinal errors occasionally exceeded 5 mm, leading the authors to recommend combining ETD with image guidance [13]. In the DIBH setting, Buschmann et al. confirmed the clinical feasibility of a combined thermo-optical and stereoscopic X-ray ETD workflow for left-sided breast DIBH, validating couch corrections against portal imaging and CBCT [14], while Tian et al. similarly reported reliable inter-fraction setup verification and intra-fraction motion monitoring with ETD in 31 patients undergoing DIBH after breast-conserving surgery [15]. Phantom-based end-to-end testing has further confirmed that the ETD DIBH breast workflow meets AAPM Task Group 302 positional tolerances [4,16]. Despite this growing evidence base, direct comparisons of ETD against conventional tattoo-based setup in routine breast-only DIBH treatment remain scarce—a gap the present study addresses.

The present study was designed to evaluate the accuracy and efficiency of SGRT using the ExacTrac Dynamic system in comparison with conventional tattoo-based setup. We focused on six-dimensional initial (pre-correction) positioning accuracy and treatment preparation time, with the aim of assessing whether SGRT can provide a reliable, efficient, and patient-centered alternative to traditional tattoo-based methods in breast cancer radiotherapy.

## 2. Materials and Methods

This cohort study was conducted between December 2022 and December 2024 at Shaare Zedek Medical Center. Patients scheduled for breast-only radiation therapy were included in the study. The study was conducted in accordance with the Declaration of Helsinki and was approved by the institutional Helsinki Committee (Institutional Review Board) of Shaare Zedek Medical Center (protocol code 0227-24-SZMC, ETD24 version 1.0, approved on 5 November 2024). All patients in both cohorts, including those with stage II–III disease, received whole-breast (breast-only) irradiation to 26 Gy in 5 fractions; no patient received regional nodal irradiation or a tumor-bed boost. All patients in both cohorts had undergone breast-conserving surgery (lumpectomy); no patient had a mastectomy. All patients in both cohorts, left- and right-sided, were treated supine in deep inspiration breath-hold (DIBH), so that breathing technique did not differ between cohorts. The same immobilization device and arm position were used in both cohorts, following the departmental standard, so that target volume and immobilization did not differ between cohorts.

The standard clinical practice for breast radiotherapy at Shaare Zedek Medical Center consists of conventional tattoo-based patient setup using room lasers for initial positioning. Patients are aligned according to permanent skin tattoos placed during CT simulation, followed by couch adjustments based on external laser alignment. Prior to each treatment fraction, daily kilovoltage (kV) imaging is performed for position verification. Orthogonal kV images are acquired with the on-board kV imaging system of the TrueBeam linear accelerator (Varian Medical Systems, Palo Alto, CA, USA) and compared with digitally reconstructed radiographs (DRRs) generated from the planning CT scan. Any detected translational or rotational deviations are corrected before treatment delivery to ensure accurate target localization. Patients treated using this workflow constituted the control group of the study.

The control cohort was randomly selected from patients treated with conventional tattoo-based setup during the study period who met the same eligibility criteria as the SGRT cohort, namely treatment site (breast only), treatment technique, and fractionation schedule (26 Gy in 5 fractions). No further matching on patient characteristics was performed. Patients in both cohorts were treated on a TrueBeam linear accelerator by the same team of radiation therapists.

Patients in the experimental group were treated using Surface Guided Radiation Therapy (SGRT) with the Brainlab ExacTrac Dynamic system (Brainlab AG, Munich, Germany). Initial patient positioning was performed using SGRT alone, without reliance on skin tattoos for daily setup. The system utilized real-time three-dimensional surface imaging to align the patient’s external anatomy with the reference surface generated from the planning CT external contour. The surface region of interest (ROI) used for registration was the ipsilateral breast and chest wall. Arm position and immobilization were identical in both cohorts, following the departmental standard.

Following SGRT-based positioning, patients underwent daily kV imaging for position verification, performed with the same TrueBeam on-board kV imaging system as in the control cohort. Orthogonal kV images were acquired and compared with DRRs from the treatment planning CT. The kV verification images served as the reference standard for assessment of setup accuracy. Any detected deviations were corrected prior to treatment delivery according to institutional protocol. The deviations analyzed in this study are the pre-correction couch shifts determined by the kV match, i.e., the residual error of the initial SGRT-based or tattoo-based setup. They therefore equal the kV corrections applied in each fraction and do not represent the final positional accuracy at the time of beam delivery. No additional verification imaging was acquired after the couch correction, so the final (post-correction) positional accuracy could not be assessed.

**Treatment workflow.** Each fraction followed the same sequence in both cohorts: (1) the patient entered the treatment room and was positioned on the couch; (2) initial setup was performed with SGRT (ExacTrac Dynamic surface guidance) in the experimental cohort, or with room lasers aligned to the skin tattoos in the control cohort; (3) orthogonal kV images were acquired and matched to the DRRs; (4) the couch shifts determined by the kV match were applied; and (5) treatment was delivered. The setup deviations analyzed in this study are those measured in step 3, that is, the difference between the patient position after initial setup (SGRT or tattoos) and the planned position, before any correction. They are therefore equal to the corrections applied in step 4. Preparation time covers steps 1–3.

Setup deviations in six dimensions (vertical, longitudinal, lateral, pitch, roll, and rotation) were recorded for all treatment fractions. Although daily setup in the experimental group was performed using SGRT alone, patients enrolled in the study also received conventional skin tattoos during CT simulation to ensure treatment continuity in the event of SGRT system failure or technical interruption. These backup tattoos were not used for daily positioning in the SGRT cohort.

Treatment preparation time was defined as the interval from patient registration in the treatment room until acquisition of the first kV verification image and was calculated from the time stamps of these two events recorded in the ARIA oncology information system (Varian Medical Systems, Palo Alto, CA, USA). The time stamps were recorded as part of routine care and were extracted retrospectively; the measurement was not blinded to the setup method. Preparation time does not include kV-based correction or beam delivery and is therefore distinct from overall treatment time. It was recorded at one treatment session per patient and was available for 48 of the 49 SGRT patients and all 20 control patients.

### 2.1. Statistical Analysis

Because each patient contributed five fractions and fractions from the same patient are correlated, the primary analysis was performed at the patient level, using a linear model with a random intercept for patient (equivalent, in this balanced design, to a comparison of patient means). Because the data were not normally distributed (Shapiro–Wilk test), effect sizes were expressed as the rank-biserial correlation (r) rather than Cohen’s d. For the six individual axes, a Bonferroni correction was applied (α = 0.05/6 = 0.0083); composite scores were treated as secondary measures. Clinically, a setup deviation of 5 mm or more was considered clinically significant. Because the study was not designed as an equivalence trial, a non-significant difference was not interpreted as evidence of equivalence. Preparation times (one measurement per patient) were compared using the Mann–Whitney U test, and a possible learning curve was assessed with Spearman correlation. Baseline characteristics were compared using the chi-square or Fisher’s exact test. Bootstrap confidence intervals, sensitivity analyses, clinical-threshold analyses, and systematic and random errors are described in the Supplementary Materials. Statistical analyses were performed using Python 3.9 (Python Software Foundation) with the scipy.stats library.

### 2.2. Data Processing

Raw positioning data were processed to calculate absolute values for accuracy analysis, while signed values were retained for directional bias assessment. Translational (cm) and rotational (degree) measurements were analyzed separately to avoid methodological errors from combining different measurement scales. Composite scores were calculated using clinical-importance weighting (translation weight = 1.0, rotation weight = 0.5) and appropriate normalization.

## 3. Results

A total of 49 patients in the SGRT cohort underwent 245 treatment fractions, with a median age of 58 years (range: 45–78), all diagnosed with breast cancer. Of these, 73.5% of cases were invasive ductal carcinoma, 8.2% were invasive lobular carcinoma, and 18.4% were ductal carcinoma in situ (DCIS). Regarding laterality, 55.1% were left-sided and 44.9% right-sided. In terms of staging, 18.4% were stage 0 (DCIS), 63.3% stage I, 12.2% stage II, and 6.1% stage III. All patients in this cohort received a total dose of 26 Gy in 5 fractions according to the FAST-Forward protocol for hypofractionated whole-breast radiotherapy [17]. The control group comprised 20 patients (100 treatment fractions), with a median age of 59 years (range: 47–80); 75% invasive ductal carcinoma, 10% invasive lobular carcinoma, and 15% DCIS; laterality was 35% left-sided and 65% right-sided; 15% were stage 0 and 85% stage I (Table 1).

**Figure 1:**
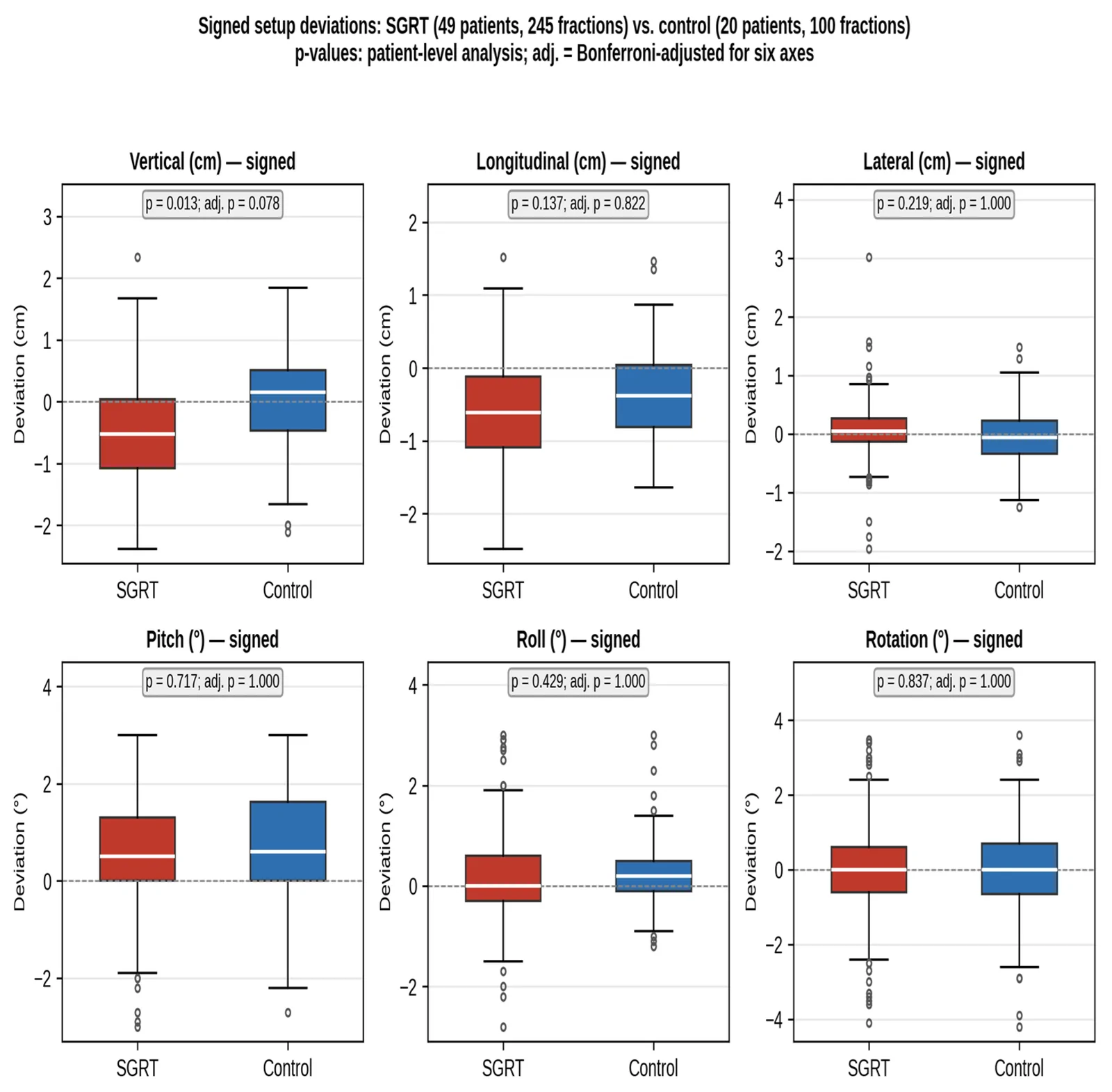
Distribution comparison of individual positioning parameters between SGRT and control cohorts (signed values). Box plots show the signed deviations for each of the six positioning parameters (vertical, longitudinal, lateral, pitch, roll, rotation) for the SGRT cohort (49 patients, 245 fractions; red) and the control cohort (20 patients, 100 fractions; blue). White lines indicate medians, boxes the interquartile range, whiskers 1.5 × IQR, and circles outliers; the dashed line marks zero. *p*-values are from the patient-level analysis; adj., Bonferroni-adjusted for six axes. Values are signed deviations; values closer to zero indicate smaller directional bias.

Setup deviations were strongly clustered within patients: the ICC of the signed deviations was 0.74–0.75 in the vertical, 0.57–0.61 in the longitudinal, and 0.16–0.31 in the lateral direction (SGRT and control, respectively), confirming that fractions from the same patient cannot be treated as independent observations (Table S5). Mean (median) signed deviations in the SGRT group were −0.48 (−0.52) cm (vertical), −0.61 (−0.61) cm (longitudinal), and +0.06 (+0.05) cm (lateral), compared with +0.03 (+0.15) cm, −0.38 (−0.38) cm, and −0.03 (−0.06) cm in the control group (Figure 1; Table S1). At the patient level, SGRT showed a vertical offset of −0.51 cm relative to control (95% CI −0.89 to −0.13 cm; *p* = 0.013), which did not remain significant after Bonferroni correction (adjusted *p* = 0.078). The longitudinal (−0.23 cm; 95% CI −0.50 to 0.05; *p* = 0.14) and lateral (+0.09 cm; *p* = 0.22) differences were not significant. Rotational deviations were small in both cohorts (mean pitch 0.65° vs. 0.74°, roll 0.16° vs. 0.25°, rotation 0.03° vs. −0.02°) and did not differ significantly (all *p* ≥ 0.43).

**Figure 2:**
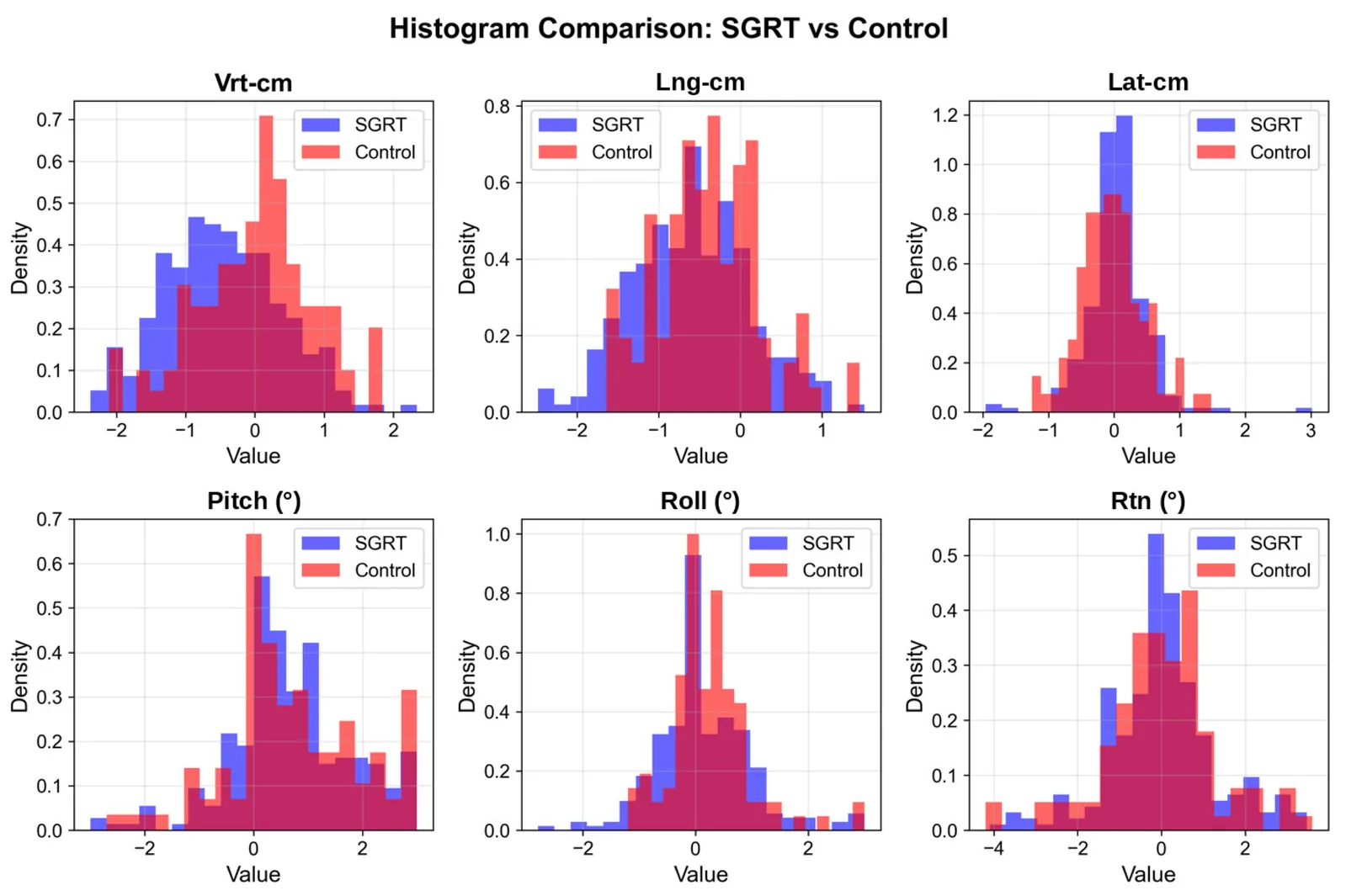
Histogram distributions of individual positioning parameters comparing SGRT and control cohorts. Density histograms show the frequency distributions of positioning errors for all six parameters. SGRT data (n = 245 fractions) are shown in blue and control data (n = 100 fractions) in red, with overlapping transparency to visualize distributional differences. All histograms are normalized to probability density to enable comparison between cohorts of different sizes. Translational parameters (Vrt-cm, Lng-cm, Lat-cm) show distinct distributional differences, with the control cohort generally showing tighter distributions around zero in the vertical and longitudinal directions; rotational parameters (pitch, roll, rotation) show more similar distributions between methods.

Box-plot and histogram analyses (Figure 1 and Figure 2) showed that the SGRT distributions were shifted in the negative direction in the vertical and longitudinal axes, with interquartile ranges similar to or slightly wider than those of the control cohort; in the lateral direction, the SGRT distribution had a narrower interquartile range but more outlying values.

**Figure 3:**
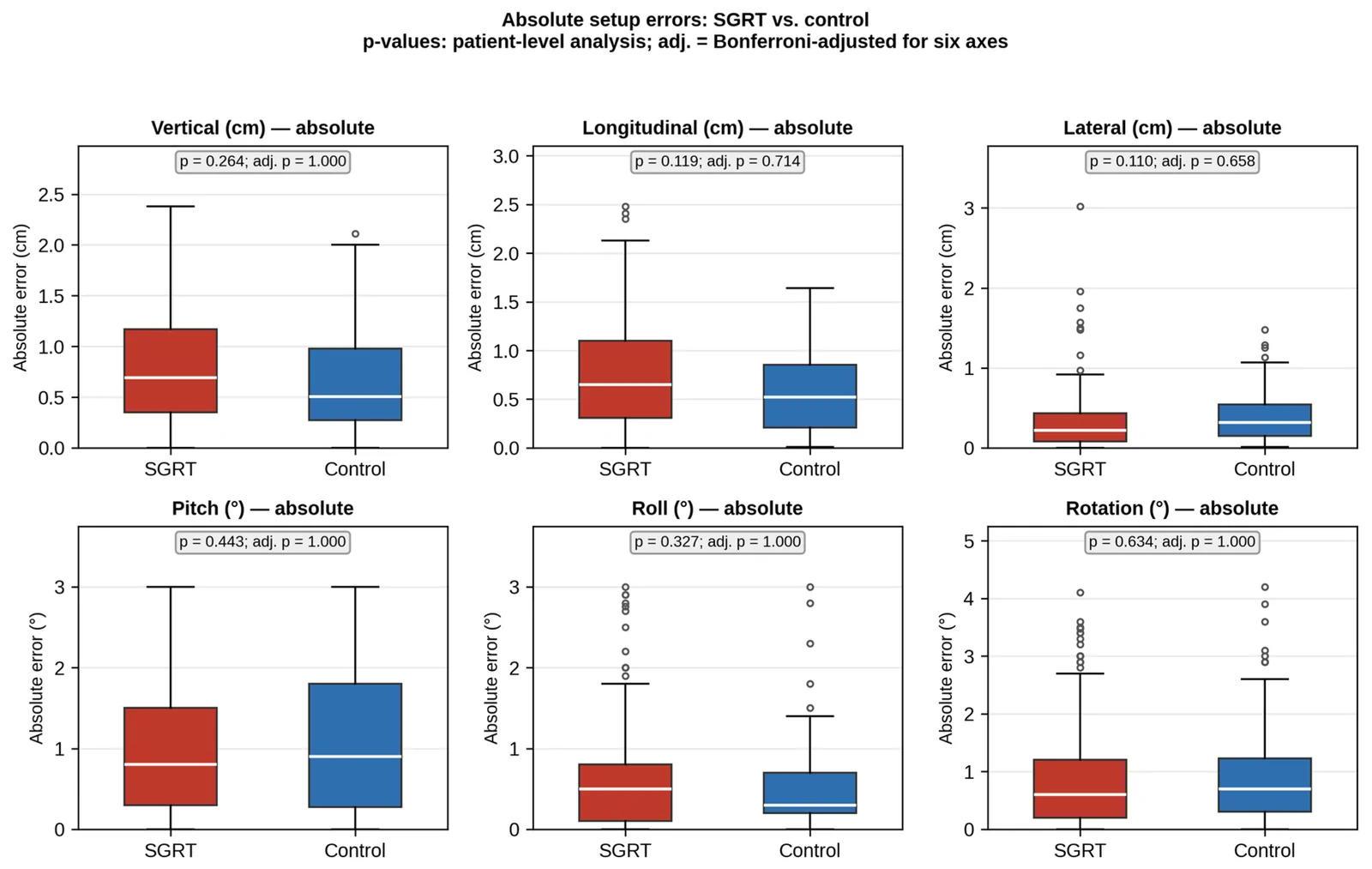
Absolute positioning errors for individual parameters comparing SGRT (red) and control (blue) cohorts. *p*-values are from the patient-level analysis; adj., Bonferroni-adjusted for six axes. No difference was statistically significant.

**Figure 4:**
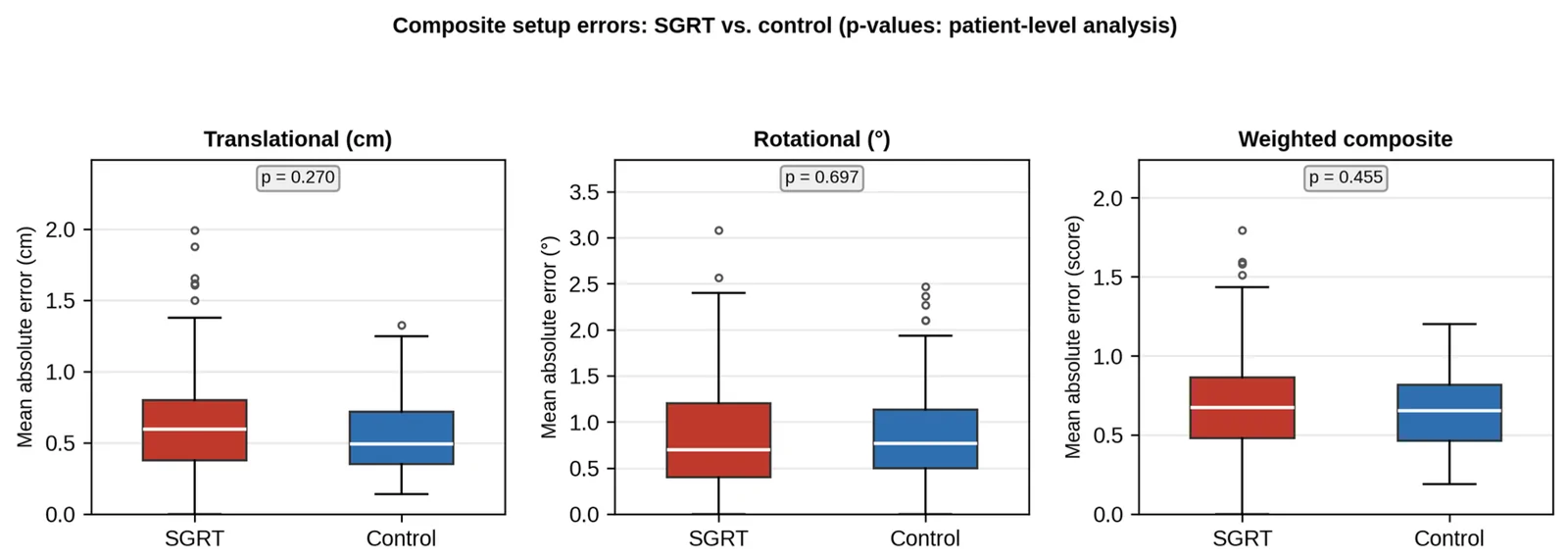
Composite positioning errors comparing SGRT (red) and control (blue) cohorts. Translational (cm) and rotational (degree) errors are summarized separately to avoid combining different units; translational and rotational accuracy represent the mean absolute error of the three translational and three rotational parameters, respectively, and the weighted composite combines them (translation weight = 1.0, rotation weight = 0.5). *p*-values are from the patient-level analysis. No difference was statistically significant; this does not by itself establish equivalence.

**Figure 5:**
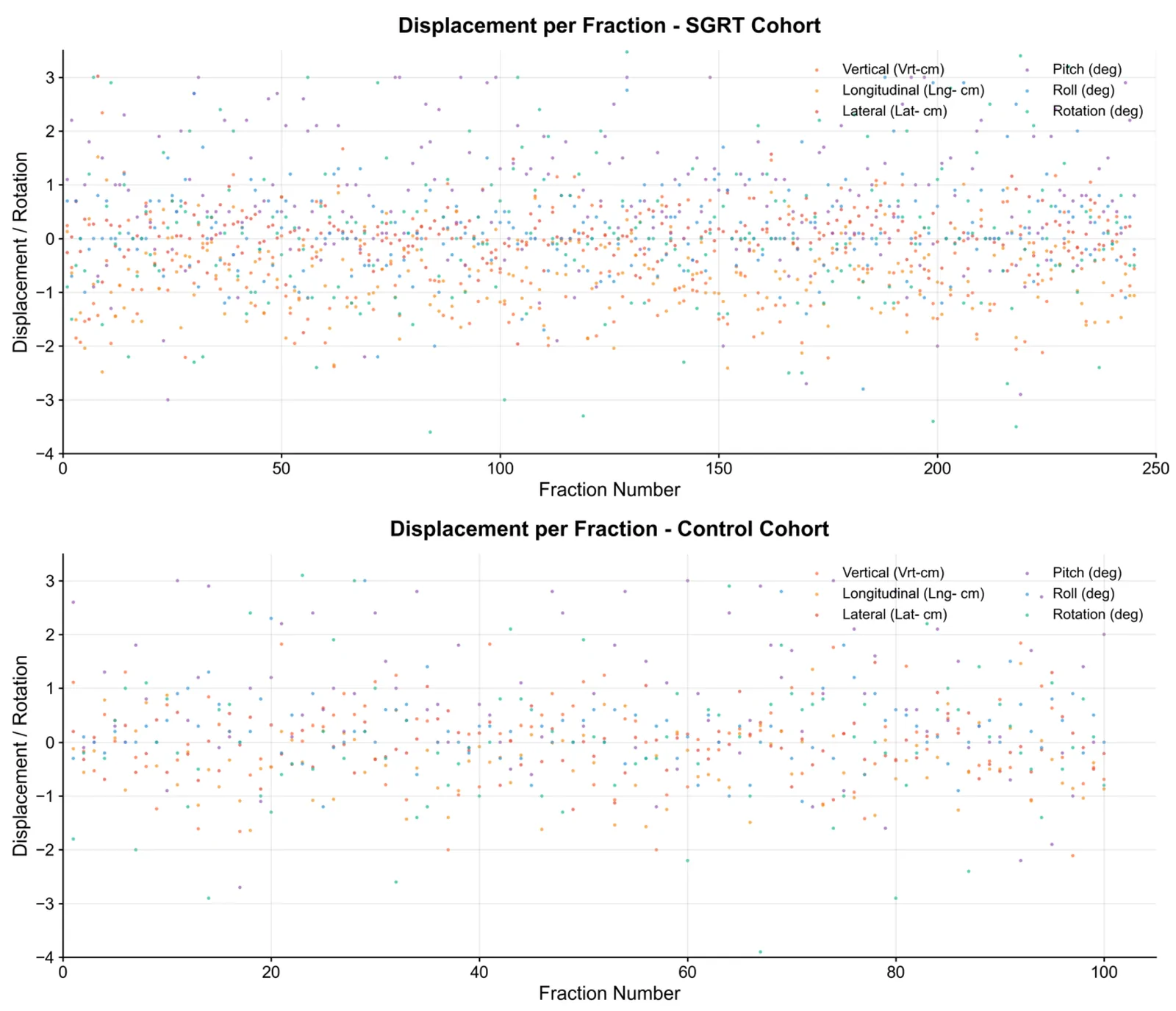
Displacement per fraction scatter plots for SGRT and control cohorts. Scatter plots display all six positioning parameters across treatment fractions for both cohorts (**upper panel**: SGRT, 245 fractions; **lower panel**: control, 100 fractions). This visualization reveals temporal patterns, variability, and outlier fractions in positioning accuracy throughout the treatment course. Both cohorts show similar overall variability, with most measurements clustered around zero and occasional outlier fractions. Translational (cm) and rotational (degree) parameters are plotted on a shared axis for visual comparison of relative variability only and are not intended for direct numerical comparison.

Absolute setup errors did not differ significantly between cohorts on any axis at the patient level (Table 2, Figure 3). Mean absolute errors were 0.79 vs. 0.66 cm (vertical; difference 0.13 cm, 95% CI −0.10 to 0.35; *p* = 0.26), 0.76 vs. 0.59 cm (longitudinal; 0.17 cm, −0.03 to 0.35; *p* = 0.12), and 0.31 vs. 0.39 cm (lateral; −0.08 cm, −0.16 to 0.01; *p* = 0.11) for SGRT and control, respectively; no difference remained significant after Bonferroni correction. The fraction-level tests used in the original analysis (vertical *p* = 0.031, longitudinal *p* = 0.013, lateral *p* = 0.004) overstated the evidence because they ignored within-patient correlation (Table S5). Effect sizes were small (rank-biserial r between −0.20 and 0.17). The standard deviations of the absolute errors were larger for SGRT than for control in all three translational directions. Composite translational (*p* = 0.27), rotational (*p* = 0.70), and weighted (*p* = 0.46) errors and the three-dimensional vector (1.25 vs. 1.11 cm; *p* = 0.29; Table S2) also did not differ significantly (Table 3, Figure 4). Scatter plots of displacement per fraction (Figure 5) showed similar stability across treatment fractions in both groups, with occasional outliers but no systematic drift.

**Clinical relevance and kV corrections.** Because the analyzed deviations are the couch corrections determined by daily kV imaging, they also describe the corrections applied. A correction was applied in 98.0% of SGRT fractions and 100% of control fractions, with a mean three-dimensional correction vector of 1.25 cm with SGRT and 1.11 cm with tattoo-based setup. Clinically significant pre-correction deviations (≥5 mm) occurred in 64.9% vs. 52.0% of fractions in the vertical direction, 64.9% vs. 53.0% in the longitudinal direction, and 20.4% vs. 31.0% in the lateral direction (SGRT vs. control), without significant differences between cohorts (Table S3). The systematic and random errors of the initial setup were similar in both cohorts; the margins that would be required without image guidance (2.5Σ + 0.7σ) [18] were about 2 cm in the vertical direction for both methods (Table S4).

**Figure 6:**
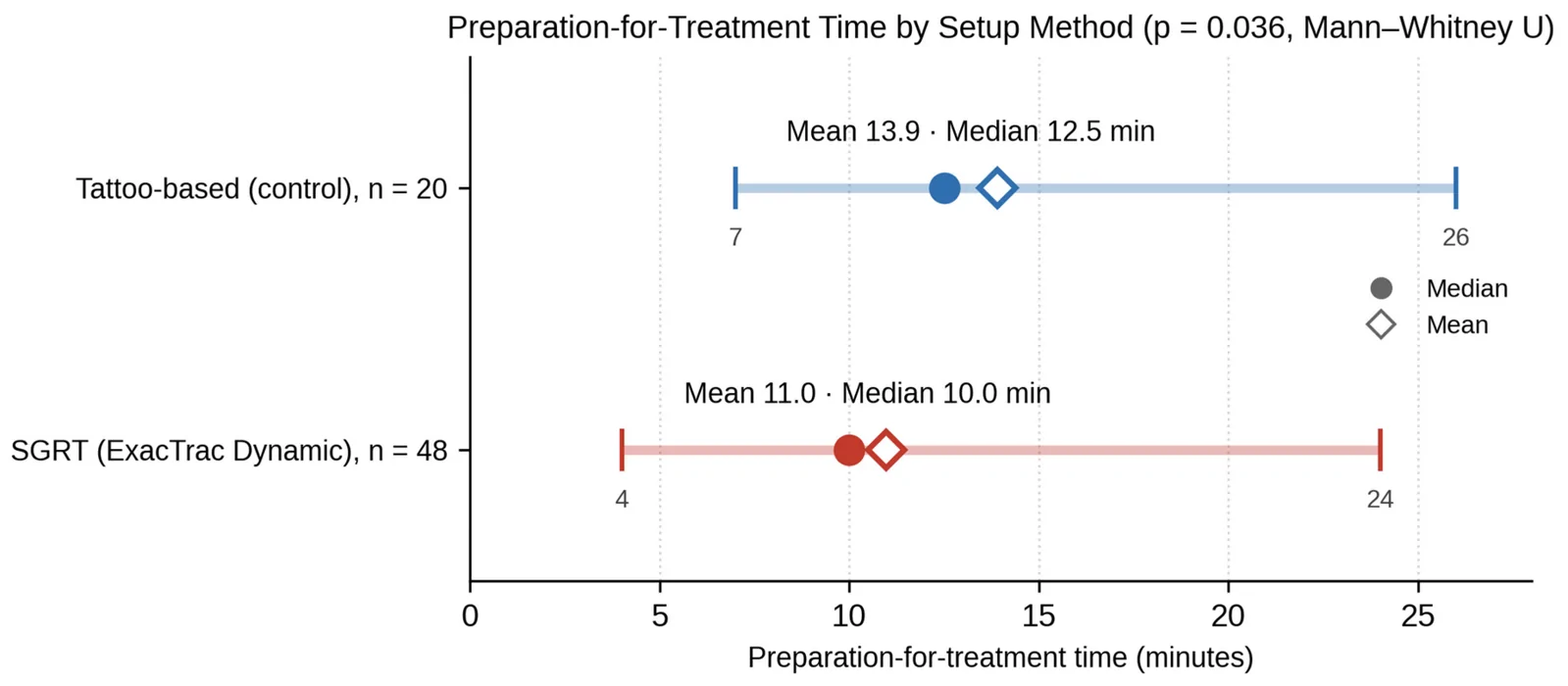
Comparison of treatment preparation time between SGRT and conventional (tattoo-based) methods. Mean (diamond) and median (circle) preparation-for-treatment time, with minimum–maximum range, for the control cohort (n = 20; mean 13.9, median 12.5, range 7–26 min) and the SGRT cohort (n = 48; mean 11.0, median 10.0, range 4–24 min); *p* = 0.036, Mann–Whitney U test. Preparation time was measured from patient registration in the treatment room to acquisition of the first kV verification image.

Preparation time was significantly shorter with SGRT: mean 11.0 min (median 10.0; range 4–24) in the SGRT cohort versus 13.9 min (median 12.5; range 7–26) in the control cohort (*p* = 0.036; Figure 6), a mean difference of approximately 3 min per treatment session. No learning curve was observed: preparation time did not correlate with the chronological order of SGRT patients (Spearman ρ = −0.11, *p* = 0.46), and median times in the first and last quartiles were similar (10.5 vs. 10.0 min, *p* = 0.28).

## 4. Discussion

This retrospective comparative cohort study found no statistically significant difference in initial setup accuracy between Surface Guided Radiation Therapy (SGRT) using the ExacTrac Dynamic system and conventional tattoo-based positioning for breast cancer radiotherapy, together with a shorter preparation time. These findings should not be read as proof of equivalence, which was not formally tested.

A key methodological point is that setup errors were strongly correlated within patients (ICC up to 0.75). An analysis that treats each fraction as an independent observation therefore overstates the effective sample size. In our original fraction-level analysis, tattoo-based setup appeared to give smaller vertical and longitudinal errors and SGRT smaller lateral errors; when patients were analyzed as the unit of observation, none of these differences remained statistically significant, and effect sizes were small. Tattoo-based setup nevertheless showed numerically smaller errors and less variability in the vertical and longitudinal directions, and SGRT showed numerically smaller lateral errors. SGRT therefore did not improve setup accuracy in our cohort.

**Systematic offset and magnitude of setup errors.** Two aspects of the positioning data require specific comment. First, the SGRT cohort showed a mean vertical offset of about −5 mm relative to the kV reference (−5.1 mm relative to the control cohort; 95% CI −8.9 to −1.3 mm), which was nominally significant but did not survive Bonferroni correction. Its magnitude, however, reaches the 5 mm level considered clinically significant. The mean longitudinal deviation was negative in both cohorts (−6.1 vs. −3.8 mm), with no significant difference between them. An offset that is consistent in direction points to a systematic rather than a random source. Several mechanisms may contribute. (i) The kV match is based on bony anatomy (chest wall, ribs, and sternum), whereas SGRT aligns the skin surface; the breast surface can move and deform relative to the underlying bone between simulation and treatment, for example, because of post-operative changes, resolution of seroma, or changes in breast shape [19]. (ii) The reference surface was derived from the planning CT external contour, and the choice of registration ROI influences the result; although our ROI included the chest wall, a large part of it consisted of the mobile ipsilateral breast, whose shape and position can change between fractions and may bias the vertical solution relative to the bony anatomy used for kV matching. (iii) Arm and shoulder position can differ slightly between days and shift the surface relative to the internal anatomy, even with identical immobilization. (iv) Differences in breath-hold depth between the planning CT and the daily treatment session displace the chest wall and breast mainly in the vertical and longitudinal directions, and may affect surface-based and bone-based registration differently. (v) A residual offset between the surface-imaging and X-ray coordinate systems cannot be fully excluded. Discrepancies between shifts detected by ETD and by volumetric imaging have also been reported by others [20], and Katayama et al. observed longitudinal errors exceeding 5 mm with ETD in free-breathing breast radiotherapy [13]. In contrast, Kügele et al. found that SGRT improved breast setup accuracy compared with laser- and tattoo-based setup [21], suggesting that such offsets depend on local implementation. Possible refinements include optimizing the ROI to emphasize the chest wall and acquiring a new reference surface after the first kV-verified fraction.

Second, pre-correction errors were large in both cohorts: mean absolute errors were 6–8 mm in the vertical and longitudinal directions, clinically significant deviations (≥5 mm) occurred in 52–65% of fractions in these directions, and the three-dimensional error exceeded 5 mm in most fractions with either method (Table S3). These values are well above the 5 mm level that we consider clinically significant. They represent initial setup errors measured before correction and reflect the difficulty of reproducing the position of the mobile breast from external references, whether tattoos or surface imaging are used. The margins that would be required without image guidance were about 2 cm vertically for both methods (Table S4). Because all deviations were corrected using daily kV imaging before beam-on in both cohorts, planning target volume (PTV) margins in this setting are determined by residual and intra-fraction errors rather than by the initial errors reported here. Our data therefore indicate that SGRT alone, like tattoo-based setup alone, is not sufficient for breast radiotherapy in our workflow, and do not support omitting daily image guidance or reducing PTV margins, in agreement with the recommendation of Katayama et al. to combine ETD with image guidance [13].

Our findings should be viewed in the context of the growing body of evidence supporting SGRT. Freislederer et al. described SGRT as an important advancement in patient positioning, demonstrating improved reproducibility and real-time motion monitoring across multiple treatment sites [22]. Similarly, Fassi et al. reported good reproducibility of the external surface position in left-breast DIBH radiotherapy [23]. Hamming et al. showed that surface imaging can position breast cancer patients in breath-hold with position errors within 5 mm while providing real-time intra-fraction monitoring [24].

Breast-specific ETD studies have reported submillimeter-to-few-millimeter geometric agreement between the integrated surface and X-ray components of ETD and volumetric imaging [12,13], and the DIBH workflow has been shown to be accurate and clinically practical [14,15]. Our study adds a direct comparison with conventional tattoo-based setup in routine breast-only DIBH treatment, in which the initial setup accuracy of ETD did not differ significantly from that of tattoos but was associated with a vertical offset.

It is important to note that most previously published SGRT studies were performed using standalone optical surface imaging systems, predominantly the AlignRT (Vision RT, London, UK) platform. These systems provide accurate surface-based patient positioning and motion monitoring but operate independently from the treatment delivery and radiographic imaging systems. In contrast, the ExacTrac Dynamic system used in the present study represents a fully integrated SGRT platform combining surface imaging with stereoscopic X-ray verification and 6-degree-of-freedom correction within a single workflow. This integration could reduce inter-system variability and streamline clinical workflows, although our data did not show an accuracy advantage for ETD over tattoo-based setup.

Beyond technical accuracy, workflow efficiency is a critical outcome in modern radiotherapy. We observed a mean reduction in preparation-for-treatment time of approximately 3 min (median 2.5 min) with SGRT, with no evidence of a learning-curve effect. This is similar to the findings of Mueller et al., who reported a median setup time of 13.0 min with tattoo-based setup versus 11.1 min with surface imaging for accelerated partial breast irradiation [2]. In our cohort, this saving was achieved without a statistically significant loss of overall accuracy, with daily kV verification maintained.

Another potential advantage of SGRT is that daily setup does not rely on permanent skin tattoos. Previous studies have highlighted the psychological burden and body-image concerns associated with radiotherapy tattoos in breast cancer patients, who may already be coping with surgical scars and other visible treatment effects [3,8]. In the present study, however, all SGRT patients also received backup tattoos at simulation, so the study demonstrates tattoo-independent daily setup rather than the elimination of tattoos. A truly tattoo-free workflow, including a fallback strategy for SGRT system failure, was not tested and should be evaluated in future work.

The integration of real-time motion monitoring with ExacTrac Dynamic may further enhance clinical safety by detecting intrafractional deviations that tattoos and static imaging cannot capture. This capability is especially valuable in advanced techniques such as DIBH, where precise breath-hold reproducibility and intra-breath-hold stability are critical to reducing cardiac and pulmonary doses in left-sided breast cancer radiotherapy [25,26]. Breath-hold reproducibility and intra-fraction motion were not analyzed in the present study.

This study has several limitations. First, it was a retrospective analysis conducted under routine clinical conditions, with a comparatively small control cohort from a single center, so that the study had limited power to detect small differences. Second, no patient-level matching was performed, and the cohorts differed in laterality (55% vs. 35% left-sided) and in stage distribution. Although surgery type (lumpectomy in all patients), target volume (breast only), fractionation, breathing technique, and immobilization were identical in both cohorts, other potential confounders, such as breast size and systemic therapy, were not available and could not be adjusted for; with 20 control patients, stratified or adjusted analyses would not have been informative. The results should therefore not be interpreted causally. Third, the study was not designed as an equivalence trial and no equivalence margin was predefined; the absence of a statistically significant difference therefore does not establish equivalence. Fourth, all SGRT patients received backup tattoos, so a fully tattoo-free workflow was not tested. Fifth, preparation time was extracted retrospectively from routine time stamps, was not blinded, and was recorded at a single session per patient. Finally, the analysis focused on initial setup errors; no verification imaging was acquired after the kV-based correction, so final positional accuracy at the time of treatment, as well as intra-fraction motion and dosimetric consequences, could not be assessed. Prospective, multicenter studies with a predefined equivalence margin are warranted.

Taken together, our study suggests that SGRT with ExacTrac Dynamic can support efficient daily setup for breast radiotherapy without relying on tattoos, with initial setup accuracy not significantly different from that of tattoo-based setup, provided that daily image guidance is maintained. The vertical offset observed in our cohort highlights the importance of careful ROI definition and reference-surface management when implementing this technique.

## 5. Conclusions

In this retrospective study, initial setup with Surface Guided Radiation Therapy (SGRT) using ExacTrac Dynamic showed no statistically significant difference in setup accuracy compared with conventional tattoo-based setup in breast cancer radiotherapy when the within-patient correlation of fractions was taken into account. SGRT was associated with a vertical offset of about 5 mm that warrants attention to reference-surface and ROI definition. Pre-correction errors were large with both methods, so daily image guidance remains necessary. SGRT shortened preparation time by approximately 3 min per treatment session (*p* = 0.036).

These results add a direct comparison with tattoo-based setup in routine breast-only DIBH treatment to the growing body of evidence supporting ExacTrac Dynamic’s accuracy and clinical feasibility previously demonstrated in DIBH and phantom-based breast studies [4,12,13,14,15,16].

Because backup tattoos were placed in all patients and equivalence was not formally tested, these findings support further evaluation of tattoo-free SGRT workflows rather than establishing them. Prospective, multicenter studies with larger patient populations, a predefined equivalence margin, and a fully tattoo-free design are warranted to validate these results and assess long-term clinical outcomes.

## Figures and Tables

**Table 1:** Baseline patient characteristics.

Characteristic	SGRT Cohort (*n* = 49)	Control Cohort (*n* = 20)	*p*-Value
Age, median (range), years	58 (45–78)	59 (47–80)	NA
Female sex, n (%)	49 (100)	20 (100)	—
Histology, n (%)			0.93
Invasive ductal carcinoma	36 (73.5)	15 (75.0)	
Invasive lobular carcinoma	4 (8.2)	2 (10.0)	
Ductal carcinoma in situ	9 (18.4)	3 (15.0)	
Laterality, n (%)			0.19
Left	27 (55.1)	7 (35.0)	
Right	22 (44.9)	13 (65.0)	
Stage, n (%)			0.25
0 (DCIS)	9 (18.4)	3 (15.0)	
I	31 (63.3)	17 (85.0)	
II	6 (12.2)	0 (0.0)	
III	3 (6.1)	0 (0.0)	
Breast-conserving surgery (lumpectomy), n (%)	49 (100)	20 (100)	—
Target volume	Breast only	Breast only	—
Regional nodal irradiation/boost	0/0	0/0	—
Breathing technique	DIBH	DIBH	—
Immobilization and arm position	Same device and position	Same device and position	—
Fractionation	5 fractions	5 fractions	—
Total dose	26 Gy	26 Gy	—

SGRT, surface-guided radiation therapy; DCIS, ductal carcinoma in situ; NA, not calculated (individual patient-level ages were not available at the time of writing). Data are n (%) unless otherwise stated. *p*-values compare cohorts using the chi-square test (histology), Fisher’s exact test (laterality), or the Fisher–Freeman–Halton exact test (stage); DCIS cases are classified as stage 0; — indicates comparison not performed.

**Table 2:** Statistical summary of individual positioning metrics using absolute values (patient-level analysis; lower values indicate smaller setup errors).

Metric	SGRT Mean ± SD	Control Mean ± SD	*p*	*p* adj.	r
Vertical (cm)	0.790 ± 0.557	0.658 ± 0.523	0.264	1.000	0.15
Longitudinal (cm)	0.761 ± 0.541	0.594 ± 0.441	0.119	0.713	0.17
Lateral (cm)	0.309 ± 0.352	0.387 ± 0.314	0.110	0.658	−0.20
Pitch (°)	0.983 ± 0.847	1.096 ± 0.935	0.443	1.000	−0.05
Roll (°)	0.584 ± 0.600	0.498 ± 0.548	0.327	1.000	0.08
Rotation (°)	0.905 ± 0.886	0.968 ± 0.936	0.634	1.000	−0.05

SGRT, 49 patients (245 fractions); control, 20 patients (100 fractions). *p*, patient-level analysis (random-intercept model, equivalent to comparison of patient means); *p* adj., Bonferroni-adjusted for six axes; r, rank-biserial correlation (positive values indicate larger errors with SGRT). No difference was statistically significant. Confidence intervals, signed deviations, and fraction-level results are given in Tables S1 and S5.

**Table 3:** Statistical summary of unit-corrected composite positioning analysis (patient-level analysis; lower values indicate smaller setup errors).

Metric	SGRT Mean ± SD	Control Mean ± SD	*p*	r
Translation (cm)	0.620 ± 0.350	0.547 ± 0.265	0.270	0.12
Rotation (°)	0.824 ± 0.539	0.854 ± 0.528	0.697	−0.04
Weighted composite	0.688 ± 0.311	0.649 ± 0.237	0.455	0.06

*p*, patient-level analysis; r, rank-biserial correlation. When positioning parameters were grouped by measurement unit, no statistically significant differences were found between SGRT and control. The three-dimensional vector and confidence intervals are given in Table S2.

## Data Availability

The data presented in this study are available on request from the corresponding author due to institutional privacy restrictions on patient-level clinical data.

## Supplementary Materials

The following supporting information can be downloaded at: https://www.mdpi.com/article/10.3390/cancers18203375/s1, Supplementary Methods: statistical analysis details; Table S1: Signed deviations and absolute setup errors by axis with bootstrap confidence intervals; Table S2: Composite setup errors and three-dimensional vector; Table S3: Proportion of fractions exceeding clinically relevant thresholds; Table S4: Systematic and random errors and margins without image guidance; Table S5: Intraclass correlation and comparison of fraction-level and patient-level analyses.
