# Supplementary material for: ExacTrac Dynamic Surface-Guided Setup Versus Tattoo-Based Positioning in Breast Cancer Radiotherapy: A Retrospective Comparison of Setup Accuracy and Preparation Time
Source: Cancers (Basel). 2026 Oct 19;18(20):3375. doi: 10.3390/cancers18203375 (PMC13702465; doi:10.3390/cancers18203375)
Supplement: Supplementary file 1 [file cancers-18-03375-s001.zip › cancers-4585436-supplementary.pdf]

Supplementary Materials

ExacTrac Dynamic Surface-Guided Setup Versus Tattoo-Based Positioning in Breast Cancer Radiotherapy: A Retrospective Comparison of Setup Accuracy and Preparation Time

B. Krayim et al.

Supplementary Methods: Statistical Analysis

Each patient contributed five fractions, and setup deviations from the same patient are correlated. The primary analysis was therefore performed at the patient level. Group differences were estimated with a linear model with a random intercept for patient; because every patient contributed exactly five fractions, this is equivalent to a comparison of patient means, which was used for inference (two-sided t-test on patient means). Ninety-five percent confidence intervals (CIs) for between-group differences were obtained with a patient-level (cluster) bootstrap, with 5000 resamples of patients within each cohort. Within-patient correlation was quantified with the intraclass correlation coefficient (ICC).

As sensitivity analyses, patient means were also compared with the Mann–Whitney U test, and p-values from the cluster bootstrap are reported; the original fraction-level Mann–Whitney U tests are shown for comparison only (Table S5). Effect sizes were expressed as the rank-biserial correlation (r) with cluster-bootstrap 95% CIs. For the six individual axes, a Bonferroni correction ($\alpha = 0.05/6 = 0.0083$) was applied separately to the signed and absolute analyses.

To assess clinical relevance, the proportion of fractions with deviations of 5 mm or more, the level considered clinically significant (per translational axis and as a three-dimensional vector, $\sqrt{(\text{vertical}^2 + \text{longitudinal}^2 + \text{lateral}^2)}$), 10 mm or more (three-dimensional vector), and 2° or more (per rotational axis) was calculated and compared between cohorts using the Mann–Whitney U test on the per-patient proportions (Table S3). Group mean (M), systematic (Σ, standard deviation of patient mean errors), and random (σ, root-mean-square of patient standard deviations) errors were calculated for each translational axis, together with the margin that would be required without image guidance, $2.5\Sigma + 0.7\sigma$ [1] (Table S4). All values are pre-correction setup errors; all deviations were corrected with daily kV imaging before treatment.

Supplementary Tables

Table S1. Signed deviations and absolute setup errors by axis: patient-level analysis with bootstrap confidence intervals.

Metric	SGRT Mean ± SD	Control Mean ± SD	Difference (95% CI)	p	p adj.	r (95% CI)
Signed deviation						
Vertical (cm)	−0.48 ± 0.84	0.03 ± 0.84	−0.51 (−0.89 to −0.13)	0.013	0.078	−0.35 (−0.60 to −0.10)
Longitudinal (cm)	−0.61 ± 0.71	−0.38 ± 0.64	−0.23 (−0.50 to 0.05)	0.137	0.822	−0.19 (−0.43 to 0.05)
Lateral (cm)	0.06 ± 0.47	−0.03 ± 0.50	0.09 (−0.07 to 0.25)	0.219	1.000	0.15 (−0.08 to 0.35)
Pitch (°)	0.65 ± 1.12	0.74 ± 1.24	−0.08 (−0.54 to 0.38)	0.717	1.000	−0.03 (−0.26 to 0.20)
Roll (°)	0.16 ± 0.82	0.25 ± 0.70	−0.10 (−0.33 to 0.11)	0.429	1.000	−0.08 (−0.24 to 0.10)

Rotation (°)	0.03 ± 1.27	−0.02 ± 1.35	0.05 (−0.45 to 0.55)	0.837	1.000	0.01 (−0.21 to 0.22)
Absolute error						
Vertical (cm)	0.790 ± 0.557	0.658 ± 0.523	0.13 (−0.10 to 0.35)	0.264	1.000	0.15 (−0.08 to 0.37)
Longitudinal (cm)	0.761 ± 0.541	0.594 ± 0.441	0.17 (−0.03 to 0.35)	0.119	0.713	0.17 (−0.06 to 0.39)
Lateral (cm)	0.309 ± 0.352	0.387 ± 0.314	−0.08 (−0.16 to 0.01)	0.110	0.658	−0.20 (−0.35 to −0.04)
Pitch (°)	0.983 ± 0.847	1.096 ± 0.935	−0.11 (−0.44 to 0.19)	0.443	1.000	−0.05 (−0.25 to 0.15)
Roll (°)	0.584 ± 0.600	0.498 ± 0.548	0.09 (−0.08 to 0.24)	0.327	1.000	0.08 (−0.09 to 0.25)
Rotation (°)	0.905 ± 0.886	0.968 ± 0.936	−0.06 (−0.37 to 0.22)	0.634	1.000	−0.05 (−0.22 to 0.13)

Mean ± SD over fractions (SGRT, 49 patients/245 fractions; control, 20 patients/100 fractions). Difference, SGRT minus control, with patient-level (cluster) bootstrap 95% CI. p, patient-level random-intercept analysis (comparison of patient means); p adj., Bonferroni-adjusted for six axes. r, rank-biserial correlation with cluster-bootstrap 95% CI (positive values indicate larger values with SGRT). For absolute errors, lower values indicate smaller setup errors.

Table S2. Composite setup errors and three-dimensional vector: patient-level analysis with bootstrap confidence intervals (lower values indicate smaller setup errors).

Metric	SGRT Mean ± SD	Control Mean ± SD	Difference (95% CI)	p	r (95% CI)
Translational (cm)	0.620 ± 0.350	0.547 ± 0.265	0.07 (−0.05 to 0.19)	0.270	0.12 (−0.11 to 0.34)
Rotational (°)	0.824 ± 0.539	0.854 ± 0.528	−0.03 (−0.20 to 0.13)	0.697	−0.04 (−0.21 to 0.12)
Weighted composite	0.688 ± 0.311	0.649 ± 0.237	0.04 (−0.06 to 0.13)	0.455	0.06 (−0.15 to 0.26)
3D vector (cm)	1.248 ± 0.685	1.108 ± 0.522	0.14 (−0.10 to 0.37)	0.290	0.12 (−0.12 to 0.34)

Difference, SGRT minus control, with patient-level (cluster) bootstrap 95% CI; p, patient-level random-intercept analysis; r, rank-biserial correlation with cluster-bootstrap 95% CI. 3D vector, $\sqrt{(\text{vertical}^2 + \text{longitudinal}^2 + \text{lateral}^2)}$.

Table S3. Proportion of fractions with pre-correction setup errors reaching clinically relevant thresholds (≥5 mm, considered clinically significant).

Threshold	SGRT, n/N (%)	Control, n/N (%)	p
Vertical ≥ 5 mm	159/245 (64.9)	52/100 (52.0)	0.141
Longitudinal ≥ 5 mm	159/245 (64.9)	53/100 (53.0)	0.134
Lateral ≥ 5 mm	50/245 (20.4)	31/100 (31.0)	0.090
3D vector ≥ 5 mm	212/245 (86.5)	91/100 (91.0)	0.422
3D vector ≥ 10 mm	150/245 (61.2)	47/100 (47.0)	0.150
Pitch ≥ 2°	40/245 (16.3)	21/100 (21.0)	0.536
Roll ≥ 2°	11/245 (4.5)	3/100 (3.0)	0.435
Rotation ≥ 2°	36/245 (14.7)	15/100 (15.0)	0.563

p, Mann–Whitney U test on the per-patient proportion of fractions exceeding the threshold (patient-level analysis). Values are pre-correction errors; all were corrected with daily kV imaging before treatment.

Table S4. Group mean (M), systematic (Σ), and random (σ) errors of the initial setup, and the corresponding margin without image guidance (cm).

Cohort	Axis	M	Σ	σ	2.5Σ + 0.7σ
SGRT	Vertical	−0.48	0.75	0.43	2.18
	Longitudinal	−0.61	0.58	0.47	1.78
	Lateral	0.06	0.27	0.43	0.97
Control	Vertical	0.03	0.77	0.43	2.21
	Longitudinal	−0.38	0.54	0.40	1.62
	Lateral	−0.03	0.34	0.42	1.13

M, mean of patient mean errors; Σ, standard deviation of patient mean errors; σ, root-mean-square of patient standard deviations; margin according to van Herk et al. [1]. These values describe the initial setup before kV correction and are not the margins used clinically, since all deviations were corrected with daily image guidance.

Table S5. Intraclass correlation (ICC) of setup errors within patients, and comparison of fraction-level and patient-level p-values.

Metric	ICC SGRT	ICC Control	p fraction-level	p patient-level (primary)	p patient-level MW	p cluster bootstrap
Vertical (signed)	0.74	0.75	<0.001	0.013	0.019	0.013
Longitudinal (signed)	0.57	0.61	0.006	0.137	0.182	0.112
Lateral (signed)	0.16	0.31	0.034	0.219	0.157	0.259
Pitch (signed)	0.42	0.49	0.664	0.717	0.817	0.725
Roll (signed)	0.19	0.18	0.262	0.429	0.479	0.403
Rotation (signed)	0.30	0.48	0.885	0.837	0.963	0.868
Vertical (absolute)	0.54	0.58	0.031	0.264	0.226	0.248
Longitudinal (absolute)	0.48	0.52	0.013	0.119	0.130	0.090
Lateral (absolute)	0.11	0.06	0.004	0.110	0.050	0.077
Pitch (absolute)	0.20	0.34	0.457	0.443	0.525	0.474
Roll (absolute)	0.15	0.11	0.223	0.327	0.165	0.278
Rotation (absolute)	0.06	0.30	0.504	0.634	0.812	0.685
Translational composite	0.45	0.52	0.073	0.270	0.253	0.202
Rotational composite	0.05	0.28	0.580	0.697	0.947	0.752
3D vector	0.46	0.56	0.084	0.290	0.299	0.225

Fraction-level, Mann–Whitney U test treating all fractions as independent (original analysis, shown for comparison only). Patient-level (primary), random-intercept model (comparison of patient means). Patient-level MW, Mann–Whitney U test on patient means. Cluster bootstrap, two-sided bootstrap p-value from 5000 patient-level resamples. Unadjusted p-values.

References

1. van Herk, M.; Remeijer, P.; Rasch, C.; Lebesque, J.V. The probability of correct target dosage: dose-population histograms for deriving treatment margins in radiotherapy. Int. J. Radiat. Oncol. Biol. Phys. 2000, 47, 1121–1135, https://doi.org/10.1016/S0360-3016(00)00518-6.
